# Expression of nitrous oxide reductase in *Paracoccus denitrificans* is regulated by oxygen and nitric oxide through FnrP and NNR

**DOI:** 10.1099/mic.0.054148-0

**Published:** 2012-03

**Authors:** Linda Bergaust, Rob J. M. van Spanning, Åsa Frostegård, Lars R. Bakken

**Affiliations:** 1 Department of Chemistry, Biotechnology and Food Sciences, Norwegian University of Life Sciences, Ås, Norway; 2 Department of Plant and Environmental Sciences, Norwegian University of Life Sciences, Ås, Norway; 3 Department of Molecular Cell Biology, Faculty of Earth and Life Science, VU University, Amsterdam, The Netherlands

## Abstract

The reductases performing the four steps of denitrification are controlled by a network of transcriptional regulators and ancillary factors responding to intra- and extracellular signals, amongst which are oxygen and N oxides (NO and ${\text{NO}}_{\text{2}}^{\text{–}}$). Although many components of the regulatory network have been identified, there are gaps in our understanding of their role(s) in controlling the expression of the various reductases, in particular the environmentally important N_2_O reductase (N_2_OR). We investigated denitrification phenotypes of *Paracoccus denitrificans* mutants deficient in: (i) regulatory proteins (three FNR-type transcriptional regulators, NarR, NNR and FnrP, and NirI, which is involved in transcription activation of the structural *nir* cluster); (ii) functional enzymes (NO reductase and N_2_OR); or (iii) ancillary factors involved in N_2_O reduction (NirX and NosX). A robotized incubation system allowed us to closely monitor changes in concentrations of oxygen and all gaseous products during the transition from oxic to anoxic respiration. Strains deficient in NO reductase were able to grow during denitrification, despite reaching micromolar concentrations of NO, but were unable to return to oxic respiration. The FnrP mutant showed linear anoxic growth in a medium with nitrate as the sole NO_x_, but exponential growth was restored by replacing nitrate with nitrite. We interpret this as nitrite limitation, suggesting dual transcriptional control of respiratory nitrate reductase (NAR) by FnrP and NarR. Mutations in either NirX or NosX did not affect the phenotype, but the double mutant lacked the potential to reduce N_2_O. Finally, we found that FnrP and NNR are alternative and equally effective inducers of N_2_OR.

## Introduction

*Paracoccus denitrificans* is a member of the α-proteobacteria, and is one of the best-characterized prokaryotes with respect to respiration. Its popularity as a model organism in the laboratory stems from the ease with which it is cultured and its genetic accessibility, as well as the resemblance of its aerobic respiratory chain to that of the mitochondrion (Richardson, 2000). In addition to the respiratory network for oxygen respiration consisting of three distinct types of oxidase (de Gier *et al.*, 1994), *P. denitrificans* expresses all four functional enzymes for denitrification; nitrate, nitrite, nitric oxide and nitrous oxide reductases (encoded by *nar*, *nir*, *nor* and *nos* gene clusters, respectively) (Zumft, 1997), allowing the complete reduction of nitrate to N_2_ under micro-oxic and anoxic conditions. This makes the organism quite flexible under fluctuating oxygen availabilities. At the same time, this flexibility requires a strict regulation, since the ATP and growth yield from oxygen respiration is significantly higher than that of denitrification (Strohm *et al.*, 2007), hence it is energetically efficient to downregulate the denitrification enzymes in the presence of oxygen. A tight coordination of the NO_x_ reductases may also be essential to avoid accumulation of the toxic intermediates NO and, to a lesser extent, ${\text{NO}}_{\text{2}}^{\text{–}}$, depending on the pH (Baumann *et al.*, 1997).

*P. denitrificans* has three known FNR paralogues for the transcriptional regulation of the denitrification machinery: FnrP, NNR and NarR (van Spanning *et al.*, 1997; Wood *et al.*, 2001). FnrP contains an oxygen-sensitive [4Fe-4S] cluster and controls the oxygen-dependent transcriptional activation of a wide range of factors, including the *nar* operon, encoding the respiratory nitrate reductase (NAR) (van Spanning *et al.*, 1997). NNR contains a haem group, is sensitive to oxygen and NO, and controls the expression of the genes encoding the nitrite (*nirS*) and nitric oxide reductases (*nor*) (Lee *et al.*, 2006; van Spanning *et al.*, 1995). The third paralogue is the nitrite/nitrate-sensitive NarR protein, which is involved in controlling nitrate reduction (Wood *et al.*, 2001).

Although many of the individual characteristics of these regulators as well as other ancillary factors involved in denitrification have been described, much remains to be discovered regarding the way that they all interact *in vivo* to create a functionally successful denitrifying phenotype. A recent paper by Bouchal *et al.* (2010) addresses this issue by describing mRNA and protein profiles in *P. denitrificans* wild-type and three mutant strains (deficient in FnrP, NNR and NarR) in response to oxygen limitation and nitrate. The results demonstrate an FnrP-controlled regulation of N_2_O reductase (N_2_OR). However, previous observations made by us indicate that FnrP is not the only transcriptional regulator of *nosZ* (unpublished data). Thus, while the main drivers of transcriptional activation of the genes encoding NAR, nitrite reductase (NIR) and nitric oxide reductase (NOR) have been identified, the exact mode of regulation of *nosZ*, encoding in many aspects the most environmentally significant enzyme in denitrification, is still somewhat unclear. In some denitrifiers the transcription of *nosZ* has been found to respond to NO, probably through factors such as DNR/DnrD/NNR (Arai *et al.*, 2003; van Spanning *et al.*, 1999; Vollack & Zumft, 2001).

In the present paper we study the role of a series of regulatory and ancillary factors during the initiation of denitrification at transition to anoxia. The effects of mutations were assessed by closely monitoring batch cultures during oxygen depletion and the onset of denitrification. This series of experiments generated detailed phenotypic datasets that supplement current understanding and finally allowed us to unveil a combined regulation of *nosZ* transcription by FnrP and NNR.

## Methods

### 

#### Bacterial strains.

This series of experiments included *P. denitrificans* wild-type (DSM413) and a number of strains with mutations in denitrification genes (*nirX*, *nirI*, *nosX*, *narR*, *fnrP*, *nosZ*, *nirX.nosX*, *nnr*, *norC*, *norB* and *fnrP.nnr*) derived from Pd1222, a rifampicin-resistant DSM413 derivative with enhanced conjugation frequency (de Vries et al., 1989). An overview of the strains is given in Table 1.

**Table 1.** Strains tested in this work All the strains included [with the exception of DSM413 and Pd92.36, in which *nirX* was deleted before insertion of a kanamycin-resistance (Km^r^) cassette in *nosX*] were insertion mutants constructed as described in Saunders *et al.* (1999) in R. J. M. v. S.’s laboratory at the Department of Molecular Cell Biology, VU University, Amsterdam, The Netherlands. All mutants were derived from Pd1222, which is a derivative of DSM413 with enhanced conjugation frequencies (de Vries *et al.*, 1989).

Strain	Relevant characteristics	Known role of protein	Source or reference
DSM413	*P. denitrificans*	Wild-type	DSM
Pd29.21	*fnrP* : : *Km^r^*	FNR/CRP-type transcriptional activator, oxygen-dependent transition to anaerobic respiration	van Spanning *et al.* (1997)
Pd77.71	*nnr* : : *Km^r^*	FNR/CRP-type transcriptional activator, controlling *nir*, *nor* and possibly *nos* expression	van Spanning *et al.* (1995)
Pd92.30	*fnrP* : : *Km^r^.nnr* truncated	Described above	van Spanning *et al.* (1997)
Pd110.21	*narR* : : *Km^r^*	FNR/CRP-type transcriptional activator controlling nitrate reduction	This study
Pd75.21	*nirI* : : *Km^r^*	Involved in transcription activation of the structural *nir* gene cluster	Saunders *et al.* (1999)
Pd102.21	*nosZ* : : *Km^r^*	N_2_OR	This study
Pd76.21	*nirX* : : *Km^r^*	Ancillary factor, involved in N_2_O reduction	Saunders *et al.* (1999)
Pd101.21	*nosX* : : *Km^r^*	Ancillary factor, involved in N_2_O reduction	Saunders *et al.* (2000)
Pd92.36	Δ*nirX nosX* : : *Km^r^*	Homologues described above	Saunders *et al.* (2000)
Pd82.21	*norB* : : *Km^r^*	Large catalytic subunit of NOR	This study
Pd81.21	*norC* : : *Km^r^*	Smaller subunit of NOR, electron transfer centre	This study

#### Batch incubation procedures.

The strains were raised from frozen stocks at 25 °C under aerobic conditions in Sistrom’s medium (Lueking *et al.*, 1978) containing rifampicin (20 µg ml^−1^) and kanamycin (25 µg ml^−1^), but no additional ${\text{NO}}_{3}^{\text{–}}$ (the Sistrom’s medium contains 17 µM ${\text{NO}}_{3}^{\text{–}}$, however). These cultures were then used as inocula for subsequent incubation experiments, which were all performed at 20 °C. In order to prevent excessively dense cultures, which would likely result in aggregation and local anoxia, the growth medium for the inocula was always half-strength. All cultures were continuously stirred at 850 r.p.m. to ensure complete dispersal of cells and proper gas exchange between liquid and headspace.

Batch incubation experiments were performed in 120 ml serum flasks containing triangular magnetic stirring bars and 50 ml full-strength Sistrom’s medium (without rifampicin and kanamycin), supplemented with 2 mM KNO_3_ unless otherwise specified. To ensure an airtight system, all flasks were crimp-sealed with rubber septa and aluminium caps. Prior to inoculation, the headspace atmospheres were replaced by helium (He)+pure oxygen by first replacing the air with pure He (repeated cycles of evacuation and He filling), then injecting oxygen to the desired concentration (7 vol% unless otherwise stated). In some cases, N_2_O was also injected in order to monitor the reduction of externally supplied N_2_O.

Two series of experiments were performed; ‘denitrification phenotypes’ and ‘N_2_OR regulation’. In ‘denitrification phenotypes’, the kinetics of denitrification during transition to anoxic conditions was characterized for each of the insertion mutants (listed in Table 1). The initial oxygen concentration was adjusted to 70 ml l^−1^ (7 vol %) before approximately 6×10^8^ cells were added through the rubber septa using sterile 1 ml syringes. Triplicate flasks were set up for each strain, and the levels of O_2_, NO, N_2_O and N_2_ were monitored by frequent sampling from the headspace (every 2 h). Since the incubation system utilized for these experiments holds only 15 stirred cultures, the phenotypic characterization of all the mutants was performed in three separate runs. One wild-type culture was included in each run. In a subsequent incubation, these incubations (‘denitrification phenotypes’) were repeated for some dysfunctional strains (NaR-, FnrP- and NNR-deficient strains), but with 2 mM ${\text{NO}}_{\text{2}}^{\text{–}}$ instead of ${\text{NO}}_{3}^{\text{–}}$.

In ‘N_2_OR regulation’ experiments, the effect of NarR, FnrP, NNR and NirI deficiency on the reduction of externally supplied N_2_O was assessed. In the first incubation experiment, culture flasks were prepared as described above, but with 20 ml N_2_O l^−1^ in the headspace (100 p.p.m.v) and the dynamics of headspace gases were monitored as above (sampling every 2 h). Because of high cell densities at oxygen depletion (7 % initial oxygen), resulting in high N_2_OR activities, gas sampling every 2 h was not sufficiently frequent to follow N_2_O dynamics in detail. Therefore, precise estimations of N_2_O reduction rates were not possible. N_2_O reduction rates were investigated further in a second incubation of wild-type and FnrP-, NNR-, NarR- and FnrP.NNR-deficient mutant strains. Each strain was exposed to two different treatments in duplicate: 0.5 % initial oxygen+1 mM KNO_2,_ and 0.5 % initial oxygen and nitrate/nitrite free medium. Sistrom’s medium contains a background of 17 µM nitrate as a Co salt. Nitrate-free Sistrom’s medium was prepared by anoxic incubations with *P. denitrificans* and subsequent filtration and autoclaving (for details of nitrate removal and N_2_O reduction assay, see Supplementary Figs S2–S8).

After inoculation, the aerobic respiration was monitored, and 1 ml pure N_2_O (~40 µmol) was added to the headspace after oxygen depletion. The N_2_OR activity was then monitored by frequent sampling (every 8 or 17 min). In order to quantify the N_2_O reduction rate per cell, the cell densities were determined by measuring the OD_660_ (*n* = OD_660_ · 1.87×10^9^ cells ml^−1^) at the time of N_2_O addition and immediately after N_2_O depletion.

#### Gas measurements.

After inoculation, cultures, blanks and gas standards were placed in a thermostatic water incubator containing a submersible magnetic stirring plate with 15 positions (Variomag HP 15, H&P Labortechnik). The cultures were continuously stirred at 850 r.p.m. to ensure full dispersion of cells and proper gas exchange between liquid and headspace. The incubator was coupled to an autosampler connected to a Varian CP 4700 micro gas chromatograph with 10 m poraPLOT U and 20 m MolSieve 5 Å columns (in parallel) each equipped with a thermal conductivity detector (TCD). Measurements of NO were performed on a Chemiluminescence NO_x_ Analyzer (Model 200A, Advanced Pollution Instrumentation). This system enabled automatic real-time monitoring of the reduction of O_2_ and accumulation of CO_2_, NO, N_2_O and N_2_ in the headspace of active bacterial cultures. The instrumentation and method are described in more detail in Molstad *et al.* (2007) and Bergaust *et al.* (2008).

## Results

### Denitrification phenotypes

We assessed the effects of a number of deficiencies (NarR, FnrP, NNR, NosZ, NirI, NirX, NorB, NorC, NosX, NirX.nosX, FnrP.NNR) on the denitrification phenotype of *P. denitrificans*. A summary of the results is presented in Table 2. The oxic growth rates (μ_oxic_) of the deficient strains were similar but differed somewhat from that of the wild-type. This was most likely not a result of the insertion mutations but rather of the slightly different characteristics of DSM413 (wild-type) and Pd1222 (carrying the mutations).

**Table 2.** Results from ‘denitrification phenotype’ experiments with nitrate as available NO_x_ (Figs 1 and 2) Mean NO maxima, oxic and anoxic growth rate constants (μ, h^−1^) were estimated based on linear regression of log-transformed N_2_ production (N_2_O accumulation in the NosZ-deficient strain and in the NirX.NosX double-deficient strain) or O_2_ reduction rates against time. +, Intact N_2_OR activity.

Strain	Maximal NO concn (nM)	μ_oxic_		μ_anoxic_		N_2_OR
		Mean	sd	Mean	sd	
Wild-type	18	0.218	0.014	0.126	0.008	+
*fnrP*	28	0.165	0.003	−*	−	+
*nnr*	50	0.163	0.002	−	−	+
*fnrP.nnr*	0.6	0.184	0.009	−	−	−
*narR*	−	0.166	0.004	−†	−	+
*nirI*	5	0.166	0.003	−	−	+
*nosZ*	19	0.172	0.008	0.090	0.005	−
*nirX*	20	0.157	0.001	0.138	0.005	+
*nosX*	17	0.164	0.007	0.144	0.009	+
*nirX.nosX*	20	0.161	0.001	0.108	0.002	−
*norB*	22 060‡	0.166	0.003	−	−	nd
*norC*	21 200‡	0.157	0.019	−	−	nd

* Anoxic growth with NO_3_ as electron acceptor was linear (see Supplementary Fig. S1). When supplied with 2 mM ${\text{NO}}_{\text{2}}^{\text{–}}$ instead of ${\text{NO}}_{3}^{\text{–}}$, the rate of N_2_ production increased exponentially; μ_anoxic_ estimated by regression was 0.11 h^−1^.

† Denitrification was restored when supplied with 2 mM ${\text{NO}}_{\text{2}}^{\text{–}}$ instead of ${\text{NO}}_{3}^{\text{–}}$; μ_anoxic_ estimated by regression was 0.09 h^−1^.

‡ NO still increasing at the end of incubation.

**Fig. 1.**
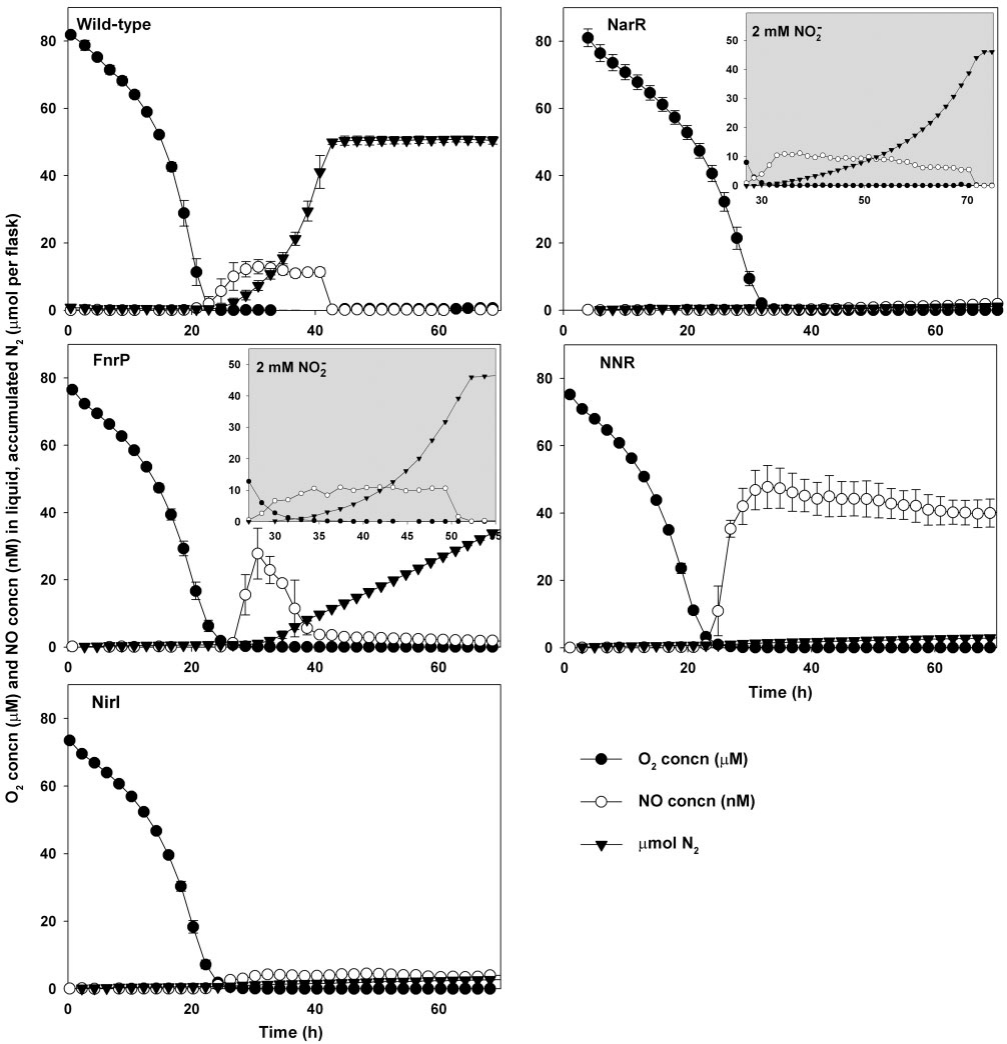
Denitrification phenotypes of wild-type and FnrP-, NirI-, NarR- and NNR-deficient mutant strains when grown in Sistrom’s medium with 2 mM nitrate (KNO_3_) and an initial O_2_ concentration of 7 vol% in the headspace. Error bars indicate sd (*n* = 3). The N_2_O concentration in the headspace remained below the detection limit (~0.5 µl l^−1^, equivalent to 2 nmol N_2_O per flask) in all cultures. The FnrP-deficient strains produced N_2_ at constant rates when grown with nitrate (main panels), but when nitrate was replaced by nitrite (2 mM KNO_2_) the rate of N_2_ production increased exponentially (insert). The NarR-, NNR- and NirI-deficient strains produced traces of N_2_ when grown with nitrate (17–20 nmol N_2_ per flask h^−1^). When supplied with nitrite, the NarR-deficient mutant produced N_2_ at an exponentially increasing rate (insert). The oxic phases of the experiments with nitrite are not shown (inserts).

Fig. 1 shows the effects of regulatory defects in mutant strains which lacked NirI or one of the three known FNR-like factors, FnrP, NarR and NNR, in comparison with the wild-type strain. These deficiencies had obvious consequences for their denitrification potential. When grown in a medium with 2 mM nitrate, the NarR-deficient mutant failed to initiate effective denitrification; the only gas production observed was a low and nearly constant rate of N_2_ accumulation (~20 nmol N_2_ per flask h^−1^). However, when nitrate in the medium was replaced by nitrite (Fig. 1, insert), the NarR-deficient strain did make the transition to denitrification, and the rate of N_2_ production increased exponentially throughout the anoxic phase, with an apparent growth rate (estimated by regression) significantly lower that that of the wild-type (0.09 versus 0.126 h^−1^).

FnrP deficiency had a less pronounced effect than NarR deficiency on the ability to reduce ${\text{NO}}_{3}^{\text{–}}$, and the culture succeeded in complete reduction of the available nitrate to N_2_. The FnrP-deficient cultures showed a pattern similar to that of the wild-type during the first hours after oxygen depletion, with an NO accumulation comparable with that of the wild-type and apparent exponential growth (as seen by N_2_ accumulation; Fig. 1). However, a few hours (~6 h) into anoxia, NO levels dropped rapidly and the rate of N_2_ production then remained constant until all nitrate was recovered as N_2_. Thus, while strains with a full set of functional FNR-type regulators and reductases completed denitrification within 17–31 h from the moment of oxygen depletion, the FnrP-deficient cultures were severely delayed, depleting nitrate only after 78 h of denitrification (100 % recovery of the available NO_3_ N as N_2_; not visible in Fig. 1 as the last 8 h of the incubation are not plotted). The conspicuously constant rate of N_2_ production by the FnrP-deficient strain was also seen upon addition of a second pulse of 2 mM nitrate (results not shown). The constant rate of denitrification does not imply that the FnrP-deficient strain was unable to grow by anoxic respiration, although the cell yield per mole of electrons to NO_x_, as deduced from final OD_660_ measurements, was lower than that of the wild-type (0.6 versus 1.7×10^13^ cells (mol *e*^−^)^−1^; see Supplementary Table S1). As for the NarR-deficient strain, the effects of FnrP deficiency were alleviated by substituting nitrate with nitrite in the medium (Fig. 1, insert). The rate of N_2_ production from ${\text{NO}}_{\text{2}}^{\text{–}}$ increased exponentially until all ${\text{NO}}_{\text{2}}^{\text{–}}$ was reduced to N_2_, although the apparent growth rate (estimated by regression) was significantly lower than that of the wild-type (0.11 versus 0.126 h^−1^; Table 2).

The strain lacking a functional NNR showed a different response. When this strain was grown in medium containing 2 mM nitrate or nitrite, the culture produced NO to a concentration of approximately 50 nM upon oxygen depletion, and this level stayed relatively constant throughout the incubation (Fig. 1). We also observed a low but constant rate of N_2_ production (single flask values ranged from 18 to 21 nmol N_2_ h^−1^). The N_2_ production was clearly above the detection limit of the system, as shown in Supplementary Fig. S9.

The NirI-deficient strain showed some similarity to the NNR-deficient strain (Fig. 1), but with a steady-state NO concentration of ~5 nM (Table 2), which was markedly lower than that of the NNR-deficient strain. As for the strain deficient in NNR, there was a low but constant rate of N_2_ production (estimates for single flasks ranged from 17 to 18 nmol N_2_ h^−1^).

**Fig. 2.**
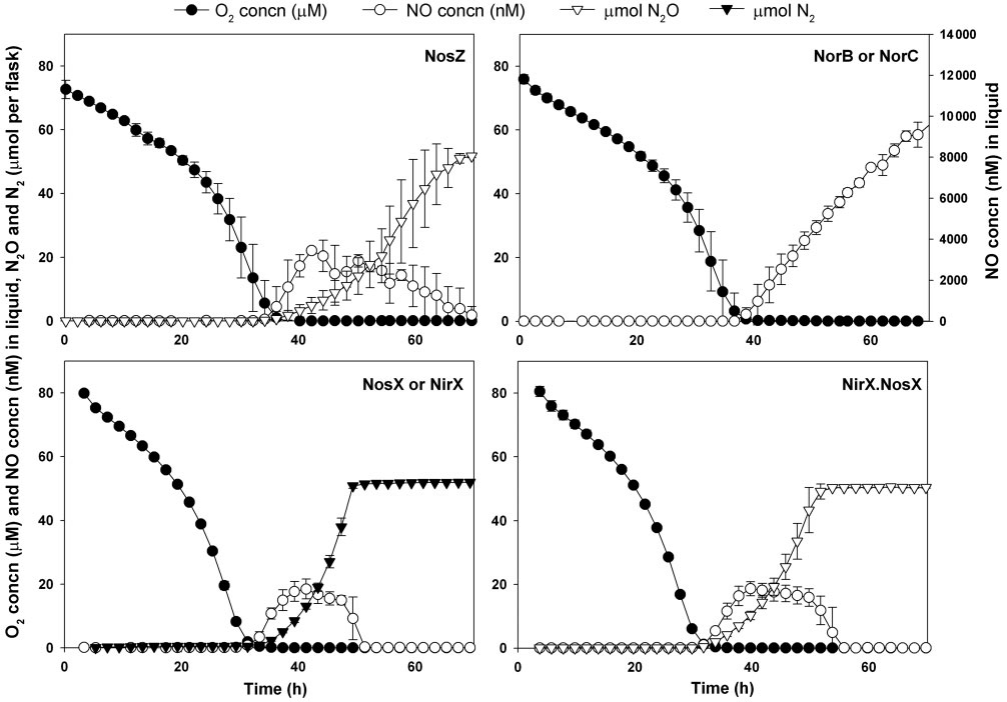
Denitrification phenotypes of NosZ-, NorB-, NorC-, NosX-, NirX- and NirX.NosX-deficient mutant strains when grown in Sistrom’s medium with 2 mM nitrate (KNO_3_) and an initial O_2_ concentration of 7 vol% in the headspace. Error bars indicate sd (*n* = 3). The results for the NorB-deficient strain were practically identical to those for the NorC-deficient strain (only one is shown). The same was the case for the NosX- and NirX-deficient strains. N_2_O remained below the detection limit (~0.5 µl l^−1^, equivalent to 2 nmol N_2_O per flask) in cultures with the NorB-, NorC-, NosX- and NirX-deficient strains. Note that the NO concentrations for NorC/NorB-deficient mutants are plotted against the right-hand *y* axis, while the left-hand *y* axis is used for the NO concentrations in the other strains.

The denitrification phenotypes of the strains with mutations in *nirX*, *nosX*, *nirX.nosX*, *norB*, *norC* and *nosZ* are summarized in Fig. 2 and Table 2. The NosZ-deficient strain lacked a functional N_2_OR and thus accumulated N_2_O as the final product of denitrification, although the kinetics of NO accumulation were similar to those of the wild-type. The kinetics of N_2_O accumulation by the NosZ-deficient strain looked similar to those of the wild-type for N_2_, although the estimated anoxic growth rate (Table 2) was significantly lower than that of the wild-type.

The strains deficient in NorC and NorB were both unable to reduce NO, with apparently lethal effects. When NO reached about 10 000 nM in cultures of these NOR mutants, the potential oxic respiration was tested in two of the replicate flasks; the headspace atmosphere was replaced by He, and oxygen was injected (0.7 ml). Neither of the mutants was able to respire the added oxygen, in contrast to the wild-type and the NosZ-deficient mutants, both of which were able to swiftly reduce a pulse of oxygen injected after depletion of ${\text{NO}}_{3}^{\text{–}}$ (data not shown). The loss of cellular integrity in NOR-deficient strains was verified further by the observation that they lacked the potential for oxic growth: the OD_660_ remained constant for 3 days despite the removal of NO and full aeration (data not shown). Despite the apparent cell death, NO continued to increase throughout the anaerobic incubation (Fig. 2), and cell numbers increased significantly (see Supplementary Table S1).

Strains deficient in either NirX or NosX easily shifted to anoxic respiration, and the dynamics of denitrification were very similar to that of wild-type. NO levels during denitrification were always below 20 nM, and the loss of either NirX or NosX was apparently of no consequence with respect to denitrification potential. The estimated anoxic growth rates were comparable with that of the wild-type (μ = 0.13±0.01 h^−1^) (Table 2). In the double mutant (mutations in both *nirX* and *nosX*), N_2_OR activity was lost and the final product of denitrification was N_2_O (Table 2, Fig. 2). As for the NosZ-deficient strain, the estimated anoxic growth rate was lower than that of the wild-type (Table 2).

### Effects of NNR, FnrP, NarR and NirI deficiencies on N_2_OR

We tested the role of NNR, FnrP, NarR and NirI in controlling the expression of N_2_OR by direct measurement of the rate at which the strains deficient in these genes were able to reduce externally supplied N_2_O. Cultures were grown under standard conditions (as shown in Figs 1 and 2), but with N_2_O in the headspace at an initial concentration of 1.8–1.9 ml l^−1^ (equivalent to 60–63 µM in the liquid, 8.3–8.8 µmol per flask). None of the strains reduced N_2_O during the early oxic phase, but when oxygen approached depletion (within the range 3–20 µM O_2_ in the liquid) the available N_2_O was rapidly reduced by all the tested single mutants. However, simultaneous deficiency in both NNR and FnrP (*fnrP.nnr* mutant) resulted in a complete loss of N_2_OR function (Table 2). The N_2_O reduction rates of wild-type and FnrP-, NNR- and NarR-deficient mutant strains were more closely assessed in a follow-up experiment using a medium with near-zero concentrations of nitrate (prepared by anoxic incubation with a small inoculum of *P. denitrificans* wild-type, then filtered and sterilized; see Methods). The results are presented in Table 3. All of the estimated mean N_2_O reduction rates fell within the narrow range of 2.33–2.89 fmol N_2_O cell^−1^ h^−1^. The double mutant (deficient in both FnrP and NNR) had no detectable N2OR activity, and the FnrP-deficient strain lost all N_2_OR activity when grown in the medium without nitrite.

**Table 3.** Mean N_2_O reduction rates±sds (fmol N_2_O per cell h^−1^) in wild-type and FnrP-, NNR-, NarR- and FnrP.NNR-deficient strains in nitrate-free medium with or without added nitrite (0 or 1 mM KNO_2_) Approximately 40 µmol N_2_O was added to cultures after oxygen depletion, and N_2_O reduction was monitored by frequent sampling from the headspace (every 8 or 17 min).

Strain	N_2_O_red_ (fmol per cell h^−1^)*	
	+${\text{NO}}_{\text{2}}^{\text{–}}$	−${\text{NO}}_{3}^{\text{–}}$
Wild-type	2.89±0.50	2.62±0.22
*fnrP^−^*	2.63±0.24	0
*nnr^−^*	2.50±0.18	2.47±0.22
*narR^−^*	2.33±0.37	2.72±0.29
*fnrP^−^.nnr^−^*	0	0

* Details are given in Supplementary Figs S2–S8.

## Discussion

In *P. denitrificans*, the FNR-type proteins FnrP, NarR and NNR are the three major players in transcriptional activation of denitrification (van Spanning *et al.*, 1997; Wood *et al.*, 2001). The expression of genes encoding nitrate reduction is under the control of both the oxygen sensor FnrP and the nitrate/nitrite sensor NarR (van Spanning, 2011). Our observation of nearly complete arrest of denitrification in the NarR-deficient strain when nitrate was the NO_x_ electron acceptor is in line with earlier observations made in *Paracoccus pantotrophus* lacking *narR* (Wood *et al.*, 2001). Denitrification was restored in *narR*^−^ cultures when grown in a medium with 2 mM ${\text{NO}}_{\text{2}}^{\text{–}}$ (instead of ${\text{NO}}_{3}^{\text{–}}$). This shows that for *P. denitrificans*, nitrate reduction is most likely the only step in denitrification regulated by NarR.

The results for the FnrP^−^ culture (Fig. 1) are less clear regarding the expression of nitrate reductase. The N_2_ production from ${\text{NO}}_{3}^{\text{–}}$ suggests that the FnrP^−^ mutant was able to express some nitrate reductase [periplasmic nitrate reductase (NAP), NAR or both] prior to complete depletion of oxygen. However, the production of nitrate reductase enzyme appeared to stop after oxygen depletion, as judged by the constant rate of N_2_ production (Fig. 1). This would imply a gradually declining amount of nitrate reductase per cell (diluted by growth) throughout the anoxic phase, which should result in linear growth. Linear growth was indeed confirmed by supplementary experiments where optical density was measured frequently (Supplementary Fig. S1). This interpretation is further strengthened by the early depletion of NO after oxygen depletion (Fig. 1), and by results with a FnrP^−^ mutant grown in a medium with 2 mM ${\text{NO}}_{\text{2}}^{\text{–}}$. In this case, the N_2_ production rate increased exponentially, and NO concentrations remained around 10 nM until depletion of ${\text{NO}}_{\text{2}}^{\text{–}}$ (Fig. 1, insert). In summary, the absence of the O_2_ sensor (FnrP) appears to eliminate expression of nitrate reductase once the conditions have shifted from micro-oxic to anoxic. The pool of nitrate reductase evidently expressed prior to complete oxygen depletion is either NAP, membrane-bound nitrate reductase (NAR) or both. It is difficult to judge which is the most likely alternative. NAP is thought to have a role in redox balancing (under oxic or micro-oxic conditions); it reduces nitrate to nitrite without conservation of energy (Gates *et al.* 2008), and its expression has been found to be low under anoxic conditions (Sears *et al.*, 2000). Thus, the observed nitrate reductase activity in the FnrP^−^ mutant could in theory be ascribed to a pool of NAP expressed prior to oxygen depletion. The relatively low cell yield per electron during anoxic growth compared with that of the wild-type lends some support to this view (no energy conservation by NAP). On the other hand, we cannot exclude the possibility that the FnrP^−^ mutant was able to express some NAR prior to oxygen depletion; van Spanning *et al.* (1997) found that FnrP in *P. denitrificans* influences NAR activity and that loss of the regulator results in a NAR activity ~30 % of that found in the wild-type. Recently FnrP deficiency was found to result in a downregulation of the β-subunit of NAR (Bouchal *et al.*, 2010). Thus, the absence of FnrP activity appears to reduce, but not eliminate, expression of NAR.

NNR is known to respond to NO, and regulates NIR and NOR expression (Saunders *et al.*, 1999; van Spanning *et al.*, 1999). Our results substantiated this role of NNR; the NNR-deficient mutant accumulated NO to reach 50 nM, which is equivalent to 70 nmol NO per flask (0.07 % of the available N in NO_3_), without any further increase (the gradual decline shown in Fig. 1 is due to dilution by sampling). This NO could in theory be ascribed to chemical decomposition of accumulating ${\text{NO}}_{\text{2}}^{\text{–}}$ (due to NAR activity), but the concentration (50 nM) is much higher than that measured in Sistrom’s medium with 2 mM ${\text{NO}}_{\text{2}}^{\text{–}}$, as illustrated in the inserted graphs in Fig. 1 (oxic phase of experiments with 2 mM ${\text{NO}}_{\text{2}}^{\text{–}}$). It appears more likely that the NO produced was due to the activity of an enzymic reduction of nitrite, by nitrate reductase (Metheringham & Cole, 1997) or other enzymes (Corker & Poole, 2003).

NirI deficiency has been shown to result in loss of *nir* transcription (Saunders *et al.*, 1999), and our results with the NirI-deficient strain are in good agreement with this: the culture accumulated a low but nearly constant amount of NO (3–4 nM) and a marginal but nearly constant production of N_2_ was observed. The constant but low production rate of N_2_ from NO_3_ by the strains lacking NirI or NNR (10–23 nmol N_2_ h^−1^, equivalent to an electron flow of 2–4×10^−18^ mol *e*^−^ per cell h^−1^) suggests a marginal pool of NIR, independent of these two transcription activators. Likewise, the similar low rate of N_2_ production from ${\text{NO}}_{3}^{\text{–}}$ by the NarR-deficient mutant suggests a marginal capacity to reduce nitrate, independent of this activator.

NO is known for its toxicity, and denitrifying bacteria are not invulnerable. As a consequence, the loss of NOR has without exception been found to be lethal under denitrifying conditions in the presence of nitrate or nitrite and intact NIR (Bergaust *et al.*, 2008; Zumft, 1997). To our knowledge, the levels of NO generated and the critical concentration causing cell death in NOR-deficient strains have not been identified. The loss of NOR did result in loss of metabolic integrity, as seen by lack of ability to respire O_2_ long before the end of the experiment. However, NO levels continued to rise throughout the incubation, indicating that the cells retained a minimum of metabolic integrity despite high concentrations of NO. The observed continued NO accumulation and increase in cell density appear to be in conflict with de Boer *et al.* (1996), who were unable to detect anoxic growth (as an increase in optical density) in the strains lacking NOR. However, their cultures were enclosed in flasks without a headspace, whereas our experiments were conducted in flasks with a 50 ml culture volume and 70 ml headspace. In our system, the production of 100 µmol NO per flask (i.e. all NO_3_ converted to NO) will result in an NO concentration in the liquid of ~70 µM, because 96 % of the NO will be in the headspace (when in equilibrium with the liquid). Without a headspace, however, the same NO production would result in 2000 µM in the liquid. This explains the contrast between the two experiments, and illustrates that denitrifying bacteria without NOR can probably grow by denitrification under natural conditions provided that the cell density is low and/or that NO can effectively escape (or be scavenged by other organisms).

Strains deficient in *nosZ*, *nirX* and *nosX*, and both *nirX* and *nosX*, were included in our experiments. NirX and NosX are periplasmic ancillary factors involved in N_2_O respiration. These proteins are orthologues and can replace each other (Saunders *et al.*, 2000). Only a *nirX.nosX* double mutation displayed a phenotype in N_2_OR function (Saunders *et al.*, 2000; Wunsch *et al.*, 2005). The nature of the effect of the double mutation on N_2_OR appears to be that the enzyme’s catalytic centre Cu_z_ remains in a redox-inert, paramagnetic state, Cu_z_* (Wunsch *et al.*, 2005), which is catalytically inactive (Dell’Acqua *et al.*, 2011). This characteristic is also found in N_2_OR isolated under aerobic conditions (Rasmussen *et al.*, 2002). Our results are in line with earlier findings. In the strains deficient in one of the factors, anoxic growth and cell yields were not very different from those of the wild-type, but in the double mutant as well as in the *nosZ*^−^ strain, the effect on N_2_OR was seen as a reduced anoxic growth rate. Wunsch *et al.* (2005) suggested a role for NosX (and consequently NirX) as a redox component in *P. denitrificans*, possibly with the membrane-bound Fe–S protein NosR as a redox partner.

The regulation of N_2_OR in *P. denitrificans* has not previously been fully resolved. In Bergaust *et al.* (2010), *nosZ* transcription was apparently induced by oxygen depletion alone, prior to any production of detectable NO, although there was a second peak in the *nosZ* transcript once NO started to accumulate. The first incubation experiments (Fig. 1) clearly showed that *P. denitrificans* was fully able to reduce N_2_O, despite FnrP deficiency. The FnrP-deficient mutant was not able to express N_2_OR when grown in a medium where all ${\text{NO}}_{3}^{\text{–}}$ had been removed, although this ability was restored by adding ${\text{NO}}_{\text{2}}^{\text{–}}$ to the medium (Table 3). The *nnr* mutant expressed N_2_OR both with and without ${\text{NO}}_{\text{2}}^{\text{–}}$, whereas the double mutant did not (Table 3). These patterns indicate that *nosZ* transcription is equally effective by an oxygen depletion signal (via FnrP) or an NO signal (via *nnr*).

The dissimilatory reduction of N oxides is orchestrated by an intricate network of genetic factors, some of which are described in the present paper. Although to some extent confirming previous knowledge, the incubation experiments performed here yielded detailed phenotypic profiles which in turn allow us to draw some new conclusions, most importantly with regard to the regulation of N_2_O reduction. N_2_OR encoded by *nosZ* is the dominant enzyme capable of reducing N_2_O to N_2_ (Zumft & Kroneck, 2007), and the understanding of its regulation and activity is thus of paramount environmental importance. The results presented here indicate a robust regulation of N_2_OR, possibly reflecting the high fitness value of swift induction and the effective reduction of the relatively inert nitrous oxide.
